# Ontario Veterinary College

**Published:** 1885-07

**Authors:** 


### ONTARIO VETERINARY COLLEGE.

The closing exercises and presentation of prizes in connection with the Ontario Veterinary College, took place in the lecture-room of the College. Dr. A. Smith, Principal, presided. Amongst the gentlemen present were the following: the Lieutenant-Governor, Col. Gzowski, Prof. Goldwin Smith, A. Blue, Deputy Minister of Agriculture; Henry Wade, Secretary, and Mr. Moore, President Agriculture and Arts Association; Dr. Thorburn, Capt. Geddes, Dr. C. J. Alloway, Montreal, and the following Examiners of the College: Wm. Cowan, Guelph; Charles Elliott, St. Catharine's; A. Coleman, Ottawa; J. H. Wilson and J. O'Neill, London; T. Lloyd, Newmarket, and C. Sweetapple, Oshawa.

In opening the proceedings, Dr. Smith said the college was instituted in 1864, and in 1866 three gentlemen graduated. Since that time there had been a regular increase in the number of graduates. The past session had been the most successful since the college was opened. They had had students from all parts of the United States and the Dominion of Canada. Those gentlemen who had graduated in former years had been successful in their practice and had commanded the respect and esteem of the communities wherever they were located. There were a number of the graduates employed with the U. S. cavalry, and others were connected with veterinary colleges and occupied prominent positions. Since last session they had lost an old and valued friend, the late Prof. Buckland. He had done a great deal to advance veterinary interests in Canada. He had given a course of lectures on breeding, and the general management of stock, etc.

The prize-list was read by Dr. Duncan, and the prizes were presented by the Lieutenant-Governor, who made a few remarks after the presentation. He said he was present two years ago, when he had listened to a most excellent account of this institution, and of the success with which it had met. He was glad to be present on this occasion and hear a still more flattering report of the progress of the college. He complimented Dr. Smith very highly in the reputation which the college had attained, as was shown by the number of students from abroad who attended it. Col. Gzowski also spoke in flattering terms of the progress made by the college, and the work it had accomplished in the interests of the profession.

### GRADUATING CLASS.

The names of the students who form the graduating class were read by Dr. Duncan as follows: John James D. Banting, Cookstown, Ont; E. R. Barnett, Smith Road, Ohio; Wm. F. Berry, Marion, Ohio; Felty A. Bolser, Newcastle, Ind; Charles Burger, Hornby, Ont; Tait S. Butler, Stirling, Ont; Jas. E. Callin, Shakespeare, Ont; Harry E. Carpenter, San Francisco, Cal; Charles Chrisman, Sharon Centre, Ohio; Eli Chrisman, Sharon Centre, Ohio; Matthew C. Crawforth, Whitby, Ont; H. E. Delavergne, Kirkland, Ill; Perry K. Dreibelbis, Lenhartsville, Pa; Thos. G. Duff, Cookstown, Ont; John E. Embury, Ingersoll, Ont; Geo. H. Farnsworth, Chester, Vt; O. D. Franks, Doylestown, Ohio; A. E. Gable, Meadville, Pa; Peter J. Gallaher, Allentown, Pa; John A. Gourlie, Summerside, P. E. I; Robert B. Grant, Dumblane, Ont; W. D. Gross, Rutztown, Pa; Frederick G. Hall, Southampton, Eng; Reuben R. Hammond, Villa Nova, Ont; W. H. Harbaugh, Cumberland, Md;

David W. Hess, Trenton, Ont; Wilson Huff, Napanee, Ont; A. L. Hunter, Hector, N. Y; J. W. Ireland, Laskay, Ont; Wm. J. Johnson, Summerville, Ont; W. J. Johnson, Croton, Ont; T. E. Jones, Granville, Ont; W. Kenney, Lindsay, Ont; J. Lawson, Almonte, Ont; J. A. Lee, Belleville, O; J. B. Lindsay, Hockley, Ont; Angus McDonald, Metcalfe, Ont; G. McGillivray, Whitby, Ont; Duncan E. McLean, Pilot Mound, Manitoba; Frank D. McMahon, Chicago, Ill; J. A. McTaggart, Milton, Ont; Archibald G. McVean, Woodbridge, Ont; F. Mathews, Northampton, Eng; Joseph N. Medill, Huston, Ont; Richard J. Michener, Waynesville, Ohio; Geo. D. Miller, Rix Mills, Ohio; Joshua Miller, Mooresville, Ont; John D. Milne, Claremont, Ont; Thos. Alex. Milne, Claremont, Ont; Wm. C. Mitchell, Tilsonburg, Ont; A. H. Moody, Grand Rapids, Mich; Geo. C. Moody, Leslie, Mich; W. W. Munger, Galesburg, Mich; C. E. Munn, Lowell, Mass; Jos. T. Natress, Woodbridge, Ont; W. D. Paxton, Fredericksburgh, Ohio; Harry B. Piatt, St. Louis, Mo; Charles H. Pierce, Grand Rapids, Mich; Samuel E. Queen, Mechanics' Town, Ohio; R. J. Quin, Edmonton, Ont; Thos. W. Scott, Duncreif, Ont; Wm. R. Shannon, Rosemont, Ont; W. S Shimer, Jr., Shimersville, Pa; Geo. Standish, Esquesing, Ont; Wm. Stevens, St. Mary's, Ont; Walter S. Tomlinson, North Henderson, Ill; Geo. Waddle, Port Dover, Ont; Alias S. Walmer, Harrisonburg, Va; Lewellyn D. Williams, Pontypridd, S. W; Wm. J. Wilson, London, Ont; Wm. Wilson, Ballymony, Antrim Co., Ireland.

---

